# Meta-Analysis and Cost Comparison of Empirical versus Pre-Emptive Antifungal Strategies in Hematologic Malignancy Patients with High-Risk Febrile Neutropenia

**DOI:** 10.1371/journal.pone.0140930

**Published:** 2015-11-10

**Authors:** Monica Fung, Jane Kim, Francisco M. Marty, Michaël Schwarzinger, Sophia Koo

**Affiliations:** 1 Beth Israel Deaconess Medical Center, Department of Internal Medicine, Boston, Massachusetts, United States of America; 2 Harvard Medical School, Boston, Massachusetts, United States of America; 3 Harvard T.H. Chan School of Public Health, Department of Health Policy and Management, Boston, Massachusetts, United States of America; 4 Brigham and Women’s Hospital, Division of Infectious Diseases, Boston, Massachusetts, United States of America; 5 Dana-Farber Cancer Institute, Boston, Massachusetts, United States of America; 6 Translational Health Economics Network, Paris, France; 7 Decision Sciences in Infectious Disease: Prevention, Control, and Care, INSERM, IAME, Paris, France; 8 Université Paris Diderot, IAME, Paris, France; Fred Hutchinson Cancer Center, UNITED STATES

## Abstract

**Background:**

Invasive fungal disease (IFD) causes significant morbidity and mortality in hematologic malignancy patients with high-risk febrile neutropenia (FN). These patients therefore often receive empirical antifungal therapy. Diagnostic test-guided pre-emptive antifungal therapy has been evaluated as an alternative treatment strategy in these patients.

**Methods:**

We conducted an electronic search for literature comparing empirical versus pre-emptive antifungal strategies in FN among adult hematologic malignancy patients. We systematically reviewed 9 studies, including randomized-controlled trials, cohort studies, and feasibility studies. Random and fixed-effect models were used to generate pooled relative risk estimates of IFD detection, IFD-related mortality, overall mortality, and rates and duration of antifungal therapy. Heterogeneity was measured via Cochran’s Q test, I^2^ statistic, and between study τ^2^. Incorporating these parameters and direct costs of drugs and diagnostic testing, we constructed a comparative costing model for the two strategies. We conducted probabilistic sensitivity analysis on pooled estimates and one-way sensitivity analyses on other key parameters with uncertain estimates.

**Results:**

Nine published studies met inclusion criteria. Compared to empirical antifungal therapy, pre-emptive strategies were associated with significantly lower antifungal exposure (RR 0.48, 95% CI 0.27–0.85) and duration without an increase in IFD-related mortality (RR 0.82, 95% CI 0.36–1.87) or overall mortality (RR 0.95, 95% CI 0.46–1.99). The pre-emptive strategy cost $324 less (95% credible interval -$291.88 to $418.65 pre-emptive compared to empirical) than the empirical approach per FN episode. However, the cost difference was influenced by relatively small changes in costs of antifungal therapy and diagnostic testing.

**Conclusions:**

Compared to empirical antifungal therapy, pre-emptive antifungal therapy in patients with high-risk FN may decrease antifungal use without increasing mortality. We demonstrate a state of economic equipoise between empirical and diagnostic-directed pre-emptive antifungal treatment strategies, influenced by small changes in cost of antifungal therapy and diagnostic testing, in the current literature. This work emphasizes the need for optimization of existing fungal diagnostic strategies, development of more efficient diagnostic strategies, and less toxic and more cost-effective antifungals.

## Introduction

Invasive fungal disease (IFD) is an important cause of morbidity and death in hematologic malignancy or hematopoietic stem-cell transplantation (HSCT) patients with high-risk febrile neutropenia (FN) [1–6]. These infections confer a substantial economic burden due to increased hospital stays and drug costs [7,8].

Prompt initiation of appropriate antifungal therapy early in the course of IFD reduces IFD-related mortality. Because of the consequences of not treating IFD early and limitations in existing IFD diagnostic methods, many patients with high-risk neutropenia and persistent or recurrent fevers despite broad-spectrum antibiotic therapy receive empiric antifungal therapy [4,9].

Early IFD diagnosis remains challenging because of the low sensitivity and specificity of clinical symptoms, microbiological cultures, and radiologic tools for IFD. Non-culture-based techniques have been developed to identify *Aspergillus* species and other fungi, including detection of circulating *Aspergillus* galactomannan [10–12] and fungal (1→3)-β-D-glucan antigens [13–15], and polymerase chain reaction (PCR) assays targeting *Aspergillus* nucleic acid sequences [16]. These tests, in conjunction with high-resolution chest computed tomography (CT), are frequently used to diagnose IFD in patients with high-risk FN [17,18].

Given these developments in IFD diagnostic testing and the toxicity and costs of prolonged antifungal exposure, diagnostic test-guided pre-emptive antifungal therapy has been evaluated as an alternative strategy to empirical antifungal therapy. Reserving antifungal therapy for the subset of patients who have early evidence of IFD by careful clinical assessments and serial fungal biomarker evaluations might reduce antifungal drug use and its attendant toxicity and costs without increasing IFD-related morbidity or mortality.

Studies of varying design have evaluated the feasibility and clinical effectiveness of empirical and pre-emptive approaches for antifungal therapy in FN patients [19–23]. Here, we use a systematic approach to summarize the existing literature on IFD outcomes and antifungal use with empirical versus pre-emptive antifungal strategies in hematologic malignancy and HSCT patients with high-risk FN, and incorporate these data into a decision analysis model to compare the relative economic burden of these two strategies.

## Methods

### Systematic Review/Meta-Analysis

We searched electronically for published or publicly presented scientific literature comparing empirical and pre-emptive strategies for treating adult hematology patients with high-risk FN [9]. Using PubMed, we searched for combinations of terms including “invasive fungal disease,” “hematologic malignancy,” “pre-emptive,” “diagnostic-directed,” “febrile neutropenia,” and “antifungal” to find published literature from January 1990 to June 2015. A manual search of the reference lists of select articles was also conducted. We searched for abstracts from major scientific meetings, including the Interscience Conference on Antimicrobial Agents and Chemotherapy, Infectious Diseases Week Conference, and the Infectious Disease Society of America (IDSA), American Society of Clinical Oncology, and American Society of Hematology Annual Meetings. For conference abstracts meeting our search criteria, posters or presentations were reviewed to facilitate data abstraction. This study is not included in a systematic review registry.

Data were extracted independently by primary researchers, and cross-checked by other investigators. Outcomes measured included IFD detection, IFD-related mortality, and rates of “pre-IFD diagnosis” empirical antifungal therapy, defined as antifungal therapy administered prior to the start of treatment for incident probable or proven IFD [24]. When these outcomes were not explicitly reported in the primary literature, we calculated these parameters using reported data. We used Mantel-Haenszel (M-H) fixed-effects and Dersimonian-Laird (D-L) random effects models to generate pooled estimates of outcomes, represented as relative risks (RR) and 95% confidence intervals (95% CI) comparing pre-emptive to empirical strategies.

We assessed heterogeneity by calculating Cochran's Q, I-squared, and the between-study variance tau-squared. Bias was assessed at the study level. Specifically, we used the Cochrane Collaboration’s “Risk of Bias” tool to assess bias in randomized controlled studies and the Newcastle-Ottawa Scale to assess for risk of biased in non-randomized studies. As observational studies are at higher risk of being biased, a restricted analysis was conducted, only including more robust randomized-controlled studies. All statistical analyses were conducted using Stata 13 software.

### Cost Comparison

We created a decision tree model (TreeAgePro 2015, TreeAge Software, Inc, Williamstown, MA) and conducted a cost analysis comparing two strategies for antifungal therapy among neutropenic patients with hematologic malignancy or HSCT at high risk for IFD: (1) an empirical (fever-directed) approach, in which patients febrile after 4 days of broad-spectrum antibiotics start empirical antifungals [9], and (2) a pre-emptive (diagnostic test-based) approach, in which patients start pre-emptive antifungal therapy if they develop a positive serum galactomannan test and pneumonia on chest imaging. Patients in both groups receive subsequent treatment antifungals if they develop incident proven or probable IFD [24]. Specifically, we report the incremental cost difference (in 2014 U.S. dollars) of pre-emptive versus empirical treatment strategies per patient with a prolonged FN episode (starting at >4 days) for (a) the pre-emptive or empirical period alone and (b) the overall treatment period, including treatment of incident proven or probable IFD.

**Fig 1:**
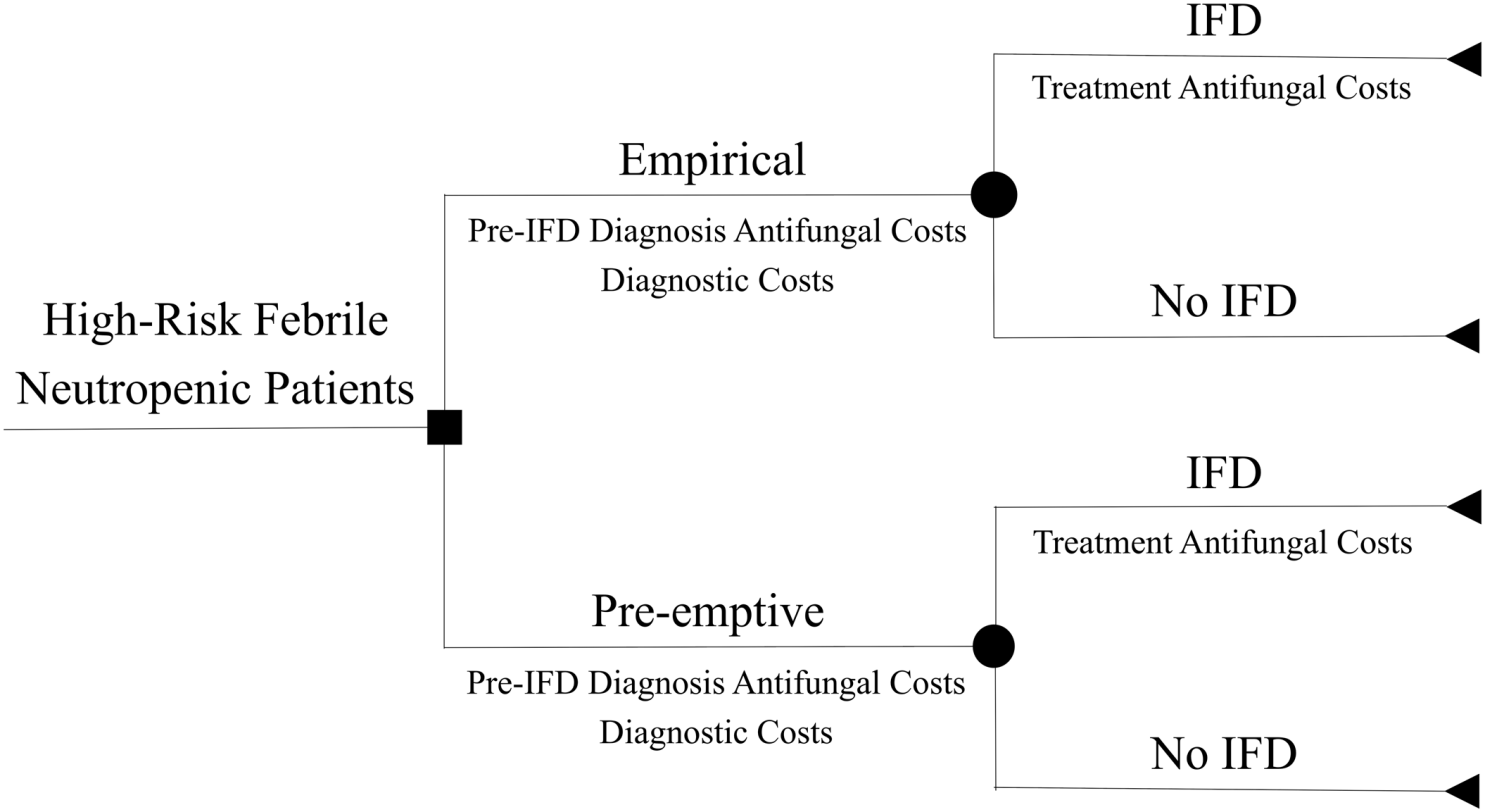
Cost comparison model of empirical versus pre-emptive antifungal therapy in high-risk neutropenic patients.

Fig 1 shows the structure of our cost comparison model. Based on our meta-analysis, we assumed no difference in overall IFD-related morbidity or mortality between the groups. We also assumed no difference in the diagnostic testing strategy but did assume a difference in the detection and duration of pre-emptive versus empirical antifungal use prior to diagnosis of incident IFD (based on pooled RR estimates from our meta-analysis).

Based on our systematic review and meta-analysis, the diagnostic testing strategy in both groups consisted of two serum galactomannan tests per week of FN beyond 4 days and one high-resolution chest CT per FN episode. Base case diagnostic testing assumptions factored in the reality that many empirical strategies incorporate diagnostic testing [20]. Neutropenia duration was assumed to be 18 days in both groups [25–28], with initiation of either empirical or pre-emptive antifungal therapy after the first 4 days.

Antifungal agents in patients with proven or probable IFD, according to 2008 European Organization for Research and Treatment of Cancer/Mycoses Study Group (EORTC/MSG) consensus definitions [24], were assumed to be the same in the two groups. The base case choice and distribution of antifungal agents for empirical and pre-emptive therapy and for treatment of proven or probable IFD were defined by IDSA treatment guidelines for treatment of invasive aspergillosis [4,9] and by a group of Infectious Diseases physicians and clinical pharmacists with expertise in caring for hematology and HSCT patients with FN at Brigham and Women’s Hospital and Dana-Farber Cancer Institute. The duration of treatment for incident IFD was defined as 12 weeks (84 days) [4,9]. Importantly, toxicity was not incorporated into this model given evidence from our systematic review showing no significant difference in amphotericin, echinocandin, or azole-related renal or hepatic toxicity between patients receiving antifungal therapy on an empirical or pre-emptive basis [29,30].

Otherwise, per the randomized trials in our systematic review [26,31], baseline characteristics of the two antifungal therapy groups—age, gender, oncologic diagnosis, and phase of therapy (induction, relapse, consolidation, and transplantation)—were assumed to be similar. We also assumed healthcare utilization and health outcomes of the two groups beyond specified differences in the antifungal therapeutic strategy and measured clinical outcomes were similar.

Direct costs for diagnostic imaging were derived from a publicly-available database on health care costs [32]. The cost of serum galactomannan testing was derived from the Brigham and Women’s Hospital and Dana-Farber Cancer Institute laboratory costs. Antifungal drug costs were based on average wholesale price from the Red Book [32].

We conducted a probabilistic sensitivity analysis (second-order Monte Carlo simulation) with 10,000 simulations and lognormal distributions on pooled RR estimates in our model to calculate 95% credible intervals for the incremental cost difference between pre-emptive and empirical antifungal strategies. We also conducted best- and worst-case analyses around diagnostic testing strategy and antifungal drug costs (using the least and most costly antifungal agents). Finally, we conducted sensitivity analyses using only parameters derived from large randomized controlled trials of empirical versus preemptive antifungal strategies.

## Results

### Systematic Review/Meta-Analysis

**Fig 2:**
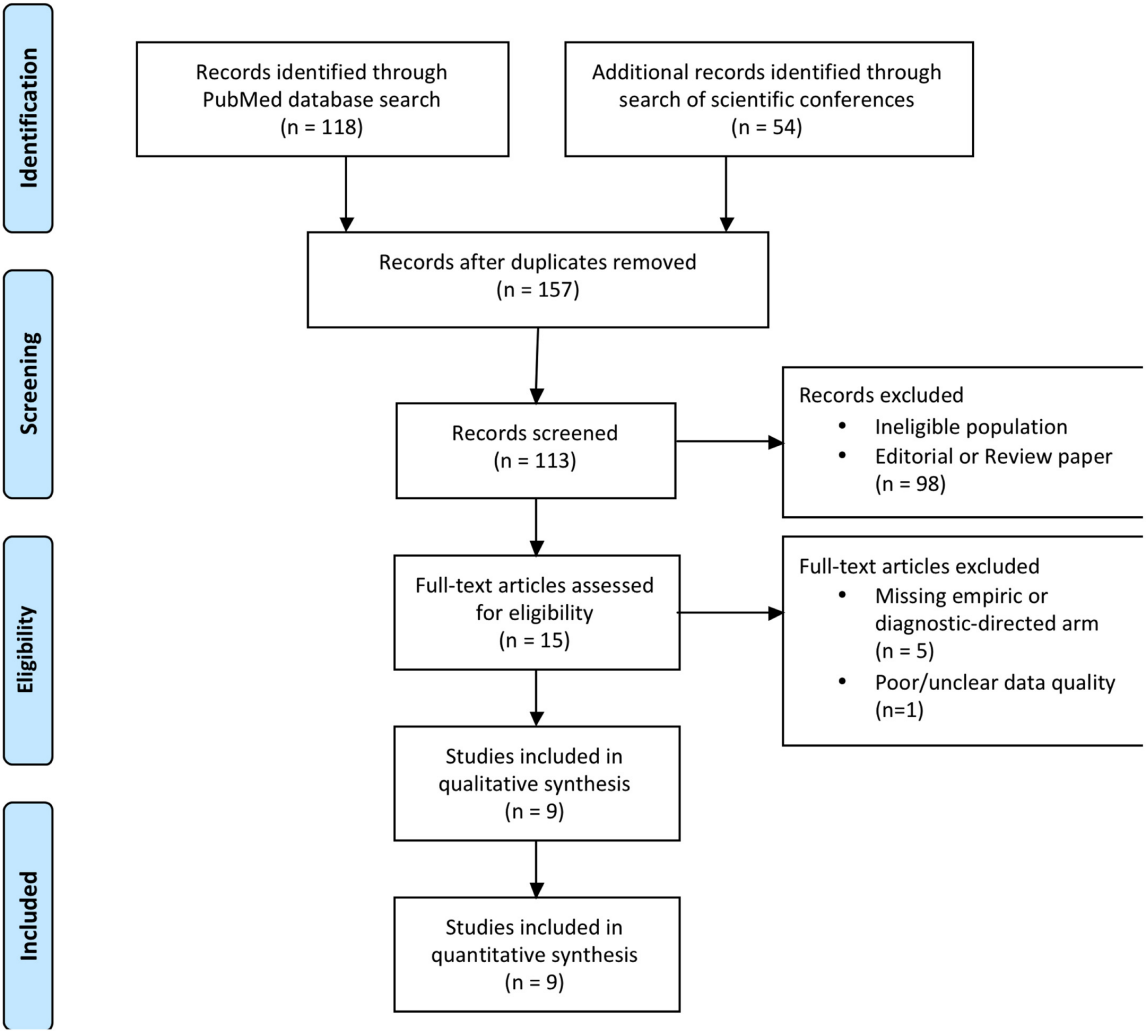
PRISMA Flow Diagram of studies included in systematic review and meta-analysis.

Our initial query returned 172 studies. Of these, ten published articles met our inclusion criteria, presenting primary literature comparing empirical and pre-emptive antifungal treatment strategy in hematologic malignancy or HSCT patients with high-risk FN. One study was further excluded due to uncertainty about data quality (Fig 2). Two presentations from infectious diseases and mycology conferences met our inclusion criteria, but both corresponded with one of the ten published articles [26,28]. Table 1 summarizes the design of these studies and Tables 2 and 3 summarize the meta-analysis results of these reports [26,28–31,34–37].

**Table 1:** Summary of studies comparing empirical versus pre-emptive antifungal therapy in high-risk febrile neutropenic patients.

Study	Design	Population	Study Period	Empirical Protocol	Pre-emptive Protocol	Diagnostic Testing	Antifungal	Primary Endpoint
Morrissey 2013 [31]	Open-label randomized controlled trial	AUSTRALIA (n = 240) Patients ≥18 years undergoing allogeneic HSCT or intensive chemotherapy for AML or ALL	9/2005-11/2009	Antifungal drug started after persistent fever for ≥3 consecutive days	Antifungal drug started after a single positive GM, single positive PCR result, or serially negative results in patients with characteristic chest CT findings	GM 2x/week; Nested *Aspergillus* PCR 2x/week; Chest CT after positive GM or PCR	AmB, L-AmB, Voriconazole per Australian consensus guidelines	Antifungal treatment within 26 weeks of enrollment
Cordonnier 2009 [26]	Open-label randomized non-inferiority trial	FRANCE (n = 293) Patients ≥18 years with hematological malignancy scheduled for chemotherapy or autologous HSCT expected to have prolonged neutropenia	4/2003–2/2006	Antifungal drug started after 4 days of persistent fever or recurrent fever after 4 days	Antifungal drug started after 4 days of persistent fever with clinical/imaging-documented pneumonia or acute sinusitis, mucositis, septic shock, skin lesion suggesting IFD, unexplained CNS symptoms, periorbital inflammation, splenic or hepatic abscess, severe diarrhea, *Aspergillus* colonization or positive serum GM	GM 2x/week; Chest X-ray followed by Chest CT	AmB, L-AmB, Caspofungin, Voriconazole	Difference in mortality 14 days after recovery of neutropenia or after 60 days of study inclusion
Hebart 2009 [29]	Open-label randomized controlled trial	EUROPE (n = 403) Patients undergoing allogeneic HSCT without amphotericin allergy or existing IFD	7/1998-6/2001	Antifungal drug started after ≥ 5 days of febrile neutropenia or detection of pulmonary infiltrate	Antifungal drug started after 1 positive PCR result or detection of pulmonary infiltrate	Non-nested *Aspergillus* and *Candida* PCR; Chest CT; Abdominal CT; Blood cultures	L-AmB	IFD Detection 100 days after transplant
Blennow 2010 [34]	Open-label randomized trial	SWEDEN (n = 99) Patients undergoing RIC-HSCT without hypersensitivity to AmB	4/2002-11/2006	No intervention	Positive PCR randomized to AmB, patients with persistent fever regardless of PCR result	Non-nested *Aspergillus* and *Candida* whole blood PCR	L-AmB	100 day survival; 1 year IFD detection; IFD risk factors
Tan 2011 [35]	Open-label randomized trial	SINGAPORE (n = 47, NE = 52) Patients ≥ 12 years with hematologic malignancy undergoing intensive consolidative chemotherapy or HSCT	6/2006-10/2007	Standard of care according to institutional guidelines, empirical antibiotics allowed if indicated	Antifungals started after two positive GM x2 and/or chest CT suggestive of IFD	GM 2x/week; Chest CT after positive GM	Caspofungin, L-AmB, AmB, Voriconazole	Proven/probable IFD
Aguilar-Guisado 2010 [36]	Prospective interventional study	SPAIN (n = 66) Patients ≥ 16 years post-chemotherapy or post-HSCT	11/2002-2/2005	Antifungal drug started in patients with sepsis, or identified foci of infection, or per clinical discretion in high-risk patients	If no identified foci of infection, chest CT, abdominal ultrasound, and blood cultures with initiation of antifungal therapy if diagnostic work-up positive	Chest X-ray; Blood cultures; Chest CT in those with abnormal Chest X-ray; Abdominal ultrasound	AmB, Voriconazole, Caspofungin	IFD detection; IFD-related mortality
Oshima 2007 [30]	Retrospective chart review	JAPAN (n = 124) Adult patients who underwent allogeneic HSCT at a university hospital	9/2002–12/2005	At the discretion of the attending	Antifungal drug started after ≥ 7 days of persistent or recurrent febrile neutropenia, positive GM, and/or infiltrates or nodules on chest X-ray or CT	GM; Beta-D glucan	AmB, Micafungin, Itraconazole, Voriconazole	Development of proven/probable early invasive aspergillosis
Girmenia 2010 [28]	Prospective feasibility study	ITALY (n = 146, NE = 220) Patients ≥ 18 years with hematologic malignancy who underwent chemotherapy or autologous HSCT and developed neutropenia ≥7 days	3/2006–2/2007	—	Antifungal drug started after positive blood culture, GM, and/or characteristic chest CT findings	3 blood cultures; GM for 3 consecutive days; Chest CT; Other microbiologic or clinical examinations as indicated	Voriconazole, AmB, L-AmB, Caspofungin, Fluconazole	Rate of patients receiving antifungal therapy
Maertens 2005 [37]	Prospective feasibility study	BELGIUM (n = 88, NE = 136) Patients ≥16 years receiving chemotherapy for acute leukemia/ MDS or undergoing myeloablative allogenic HSCT	1/2003-1/2004	—	Antifungal treatment after 2+ consecutive positive galactomannan or with CT findings suggestive of IFD	GM; Chest X-ray 1-2x/week; Blood, sputum, urine, stool cultures	L-AmB	Rate of antifungal use; IFD cases

AML: Acute myelogenous leukemia, ALL: Acute lymphocytic leukemia, NE: Febrile neutropenic episodes, IFD: invasive fungal disease, HSCT: Hematopoietic stem-cell transplantation, RIC: reduced intensity conditioning, GM: *Aspergillus* galactomannan, CT: Computed tomography scan, PCR: Polymerase chain reaction, AmB: amphotericin B deoxycholate, L-AmB: liposomal amphotericin B

**Table 2:** Comparison of IFD-related outcomes in empiric versus pre-emptive antifungal therapy in high-risk neutropenic patients.

	IFD Detection			IFD-related Mortality			Overall Mortality		
Study	RR (95%CI)	Empiric (%)	Pre-emptive (%)	RR (95%CI)	Empiric (%)	Pre-emptive (%)	RR (95%CI)	Empiric (%)	Pre-emptive (%)
Morrissey 2013 [31]	4.76 (1.87–12.10)	4.1 (5/122)	19.5 (23/118)	0.86 (0.27–2.75)	4.9 (6/122)	4.2 (5/118)	1.55 (0.66–3.66)	6.6 (8/122)	10.2 (12/118)
Cordonnier 2009 [26]	3.41 (1.14–10.21)	2.7 (4/150)	9.1 (13/143)	7.34 (0.38–140.86)	0.0 (0/150)	2.1 (3/143)	1.84 (0.55–6.14)	2.7 (4/150)	4.9 (7/143)
Hebart 2009 [29]	0.99 (0.52–1.91)	8.1 (17/207)	8.2 (16/196)	0.82 (0.36–1.87)	4.8 (10/207)	3.6 (7/196)	0.99 (0.64–1.55)	16.4 (34/207)	16.3 (32/196)
Blennow 2010 [34]	—	0.0 (0/8)	7.7 (1/13)	—	—	—	—	—	—
Tan 2011 [35]	0.62 (0.11–3.39)	12.0 (3/25)	7.4 (2/27)	—	—	—	—	—	—
Aguilar-Guisado 2010 [36]	0.09 (0.01–1.75)	11.5 (3/26)	0.0 (0/40)	0.13 (0.01–2.64)	8.0 (2/26)	0.0 (0/40)	0.16 (0.04–0.71)	30.7 (8/26)	5.0 (2/40)
Oshima 2007 [30]	—	0.0 (0/13)	3.3 (2/60)	—	0.0 (0/13)	0.0 (0/60)	—	—	—
Girmenia 2010 [28]	—	—	—	—	—	—	—	—	—
Maertens 2005 [37]	—	—	—	—	—	—	—	—	—
**M-H RR (95%CI)**	**1.70 (1.12–2.57)**			0.85 (0.45–1.62)			0.99 (0.70–1.40)		
**D-L RR (95%CI)**	1.47 (0.55–3.96)			0.82 (0.36–1.87)			0.95 (0.46–1.99)		
	Q = 13.90 (df = 4), p = 0.01			Q = 3.62 (df = 3), p = 0.31			Q = 7.88 (df = 3), p = 0.05		
**Heterogeneity**	I^2^ = 71.3%			I^2^ = 17.0%			I^2^ = 61.9%		
	Between study τ^2^ = 0.81			Between study τ^2^ = 0.13			Between study τ^2^ = 0.33		

RR: relative risk, CI: confidence interval, IFD: invasive fungal disease, RR: relative risk, CI: confidence interval, M-H: Mantel-Haenszel fixed effects model, D-L: Dersimonian-Laird random effects models,—data unavailable and cannot be derived from this study

**Table 3:** Comparison of antifungal use in empiric versus pre-emptive antifungal therapy in high-risk neutropenic patients.

	Antifungal Use			Antifungal Duration (mean)		
Study	RR (95%CI)	Empiric (%)	Pre-emptive (%)	Empiric (%)	Pre-emptive (%)	p
Morrissey 2013 [31]	0.48 (0.29–0.79)	30.3 (39/122)	23.7 (18/118)	—	—	—
Cordonnier 2009 [26]	0.64 (0.50–0.81)	61.3 (92/150)	39.2 (56/143)	7.0 days	4.5 days	**<0.01**
Hebart 2009 [29]	1.56 (1.25–1.93)	36.7 (76/207)	57.1 (112/196)	84.2% (64/76) <30 days	79.5% (89/112) <30 days	NS
Blennow 2010 [34]	—	—	—	—	—	**—**
Tan 2011 [35]	0.76 (0.38–1.51)	44.0 (11/25)	33.3 (9/27)	—	—	—
Aguilar-Guisado 2010 [36]	—	—	—	—	—	**—**
Oshima 2007 [30]	0.08 (0.03–0.19)	100.0 (13/13)	6.7(4/60)	—	—	—
Girmenia 2010 [28]	0.57 (0.42–0.77)	52.8 (84/220)	30.1 (48/220)	—	—	—
Maertens 2005 [37]	0.22 (0.11–0.43)	35.0 (41/117)	7.7 (9/117)	—	—	—
**M-H RR (95%CI)**	**0.72 (0.63–0.81)**			—		
**D-L RR (95%CI)**	**0.48 (0.27–0.85)**			—		
	Q = 91.01 (df = 6), p ≤0.01					
**Heterogeneity**	I^2^ = 93.4%			—		
	Between study τ^2^ = 0.503					

RR: relative risk, CI: confidence interval, IFD: invasive fungal disease, RR: relative risk, CI: confidence interval, M-H: Mantel-Haenszel fixed effects model, D-L: Dersimonian-Laird random effects models,—data unavailable and cannot be derived from this study

**Fig 3:**
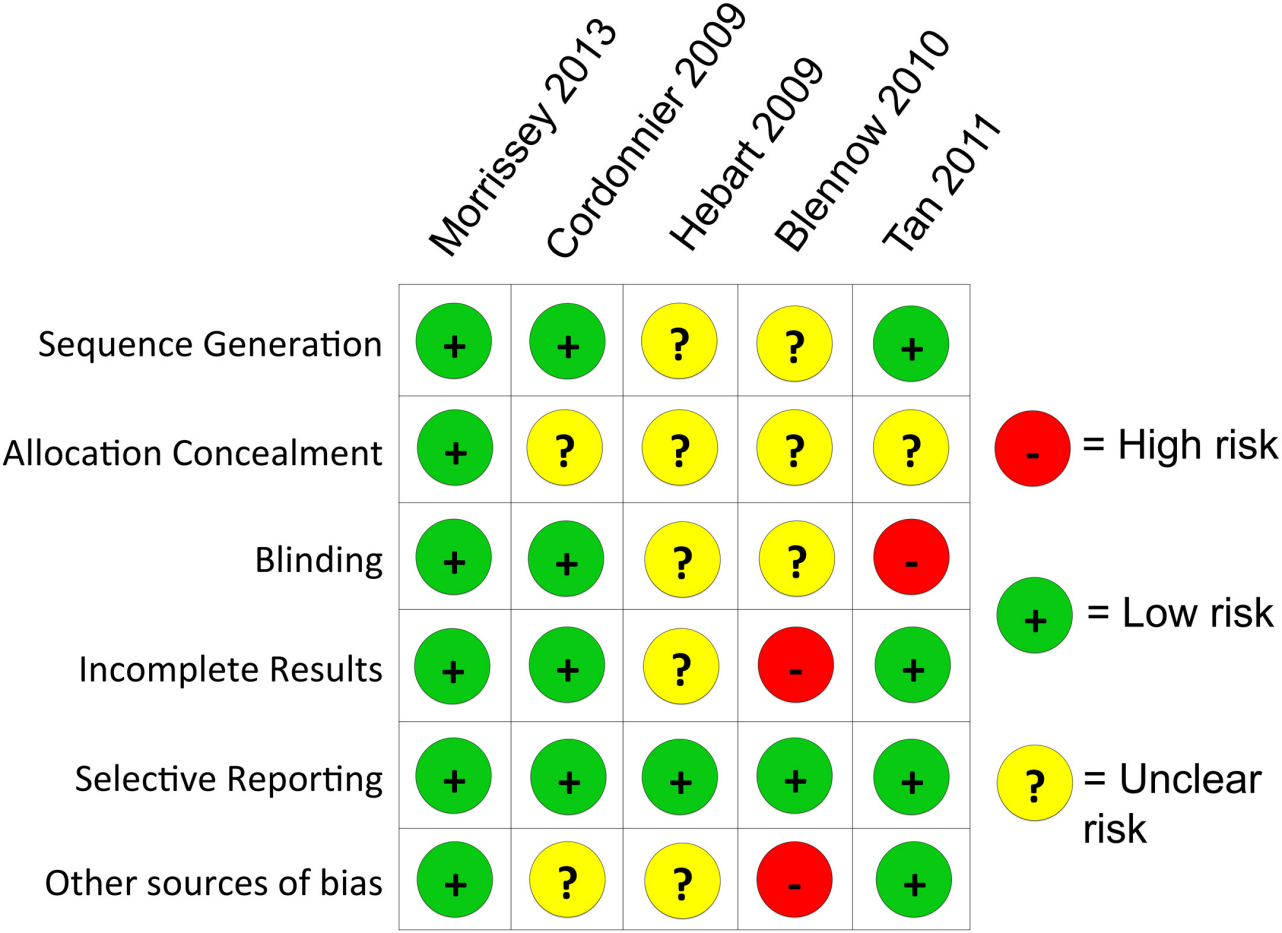
Risk of bias in randomized studies as assessed by the Cochrane Collaboration’s “Risk of Bias” tool.

**Fig 4:**
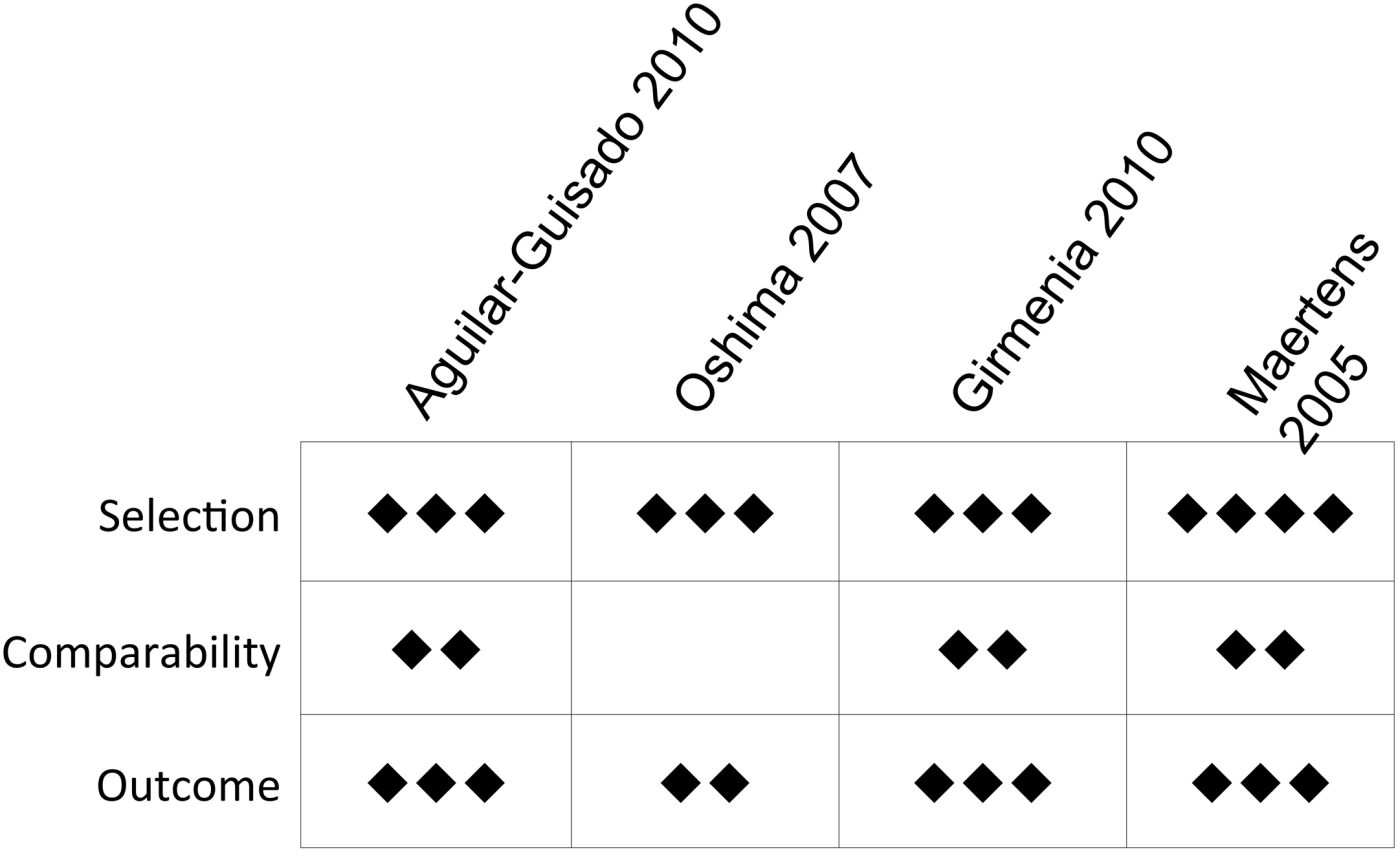
Risk of bias in non-randomized studies as assessed by the Newcastle-Ottawa Scale.

Of the nine reports meeting our inclusion criteria, five were randomized trials, two were prospective feasibility studies, one was a prospective interventional study, and one was a retrospective cohort study. Studies were published or presented between 2005–2013, with data collected from 1998–2009. Our assessments of the risk of bias are presented in Figs 3 and 4. Despite varying geographic sites, the patient populations of these studies included adults with hematologic malignancy undergoing intensive chemotherapy or HSCT

Included studies consistently defined empirical antifungal therapy as the initiation of antifungal drugs after a defined number of consecutive days with persistent febrile neutropenia. However, there was significant variation in the trigger (clinical symptoms, chest imaging, galactomannan, PCR, traditional fungal cultures) for starting antifungal therapy in the pre-emptive study groups, presenting another type of bias. The majority (9 of 10) incorporated chest imaging findings into the trigger. The remaining study [34] used a positive fungal PCR as the only trigger for initiating pre-emptive therapy. Fungal biomarkers, either galactomannan or fungal PCR, were also consistently employed in all studies as part of the pre-emptive approach, although there was variability in whether one or both were applied [26,31].

**Fig 5:**
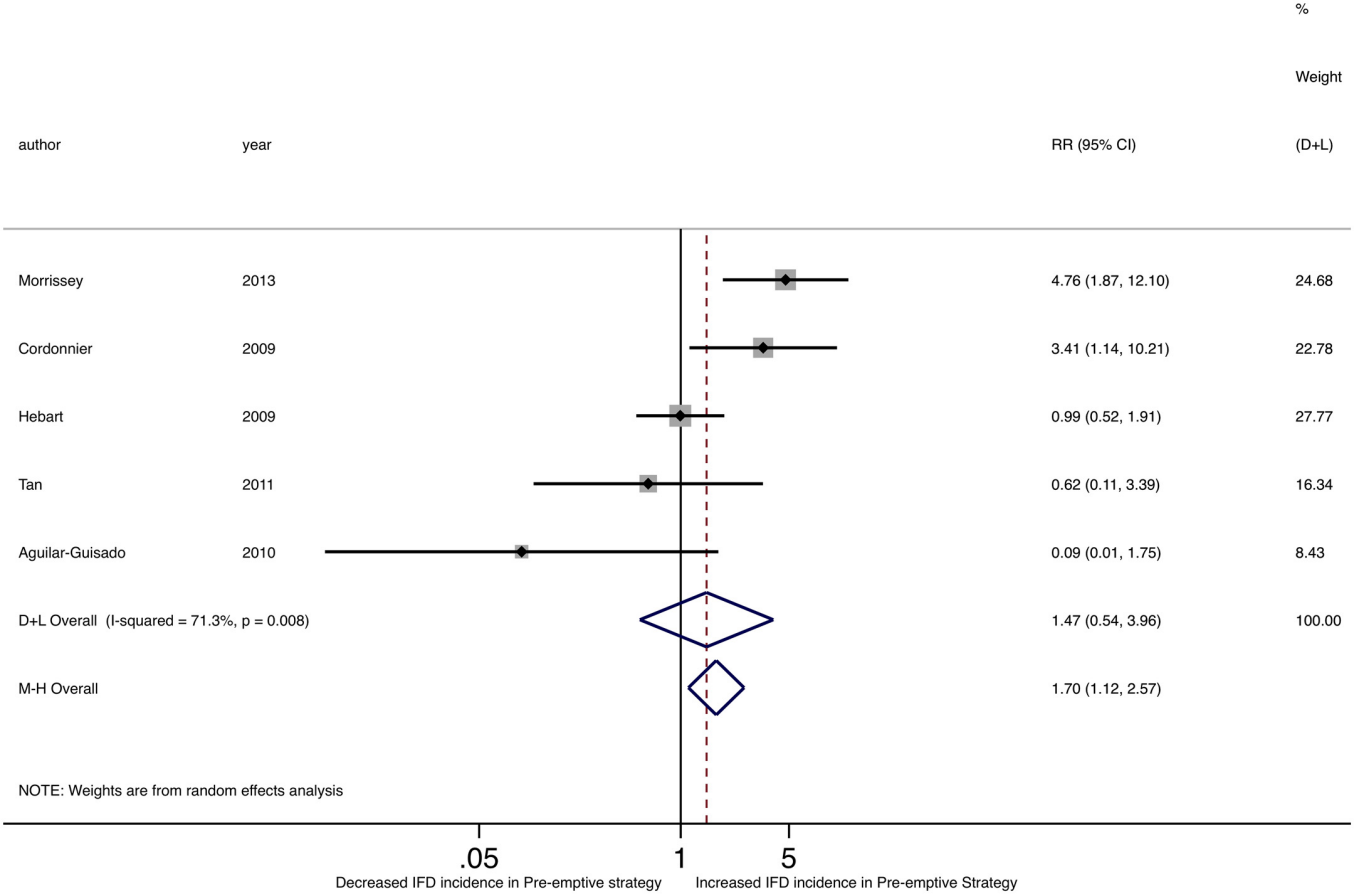
Forest plot of pooled relative risk of IFD detection comparing pre-emptive to empirical strategies.

Of seven studies that measured and reported IFD detection, two found a significantly higher rate of IFD detection in the pre-emptive therapy group (Table 2) [26, 31]. In the trial by Morrisey and colleagues, for example, IFD detection rate was 4.1% with empirical and 19.5% with pre-emptive management. Compared to studies that did not find a significant difference in IFD detection between the two antifungal strategies, those that did were better powered to identify such a difference with larger sample sizes. Pooled RR for IFD detection comparing pre-emptive to empirical strategies was 1.70 (95%CI 1.12–2.57) using a M-H fixed-effects model and 1.47 (95%CI 0.55–3.96) with a D-L random-effects model, suggesting potentially increased IFD detection with pre-emptive strategies (Fig 5). Of note, there was significant heterogeneity between studies in IFD detection (Cochran’s Q p = 0.008; I^2^ = 71.3%; τ^2^ = 0.810).

**Fig 6:**
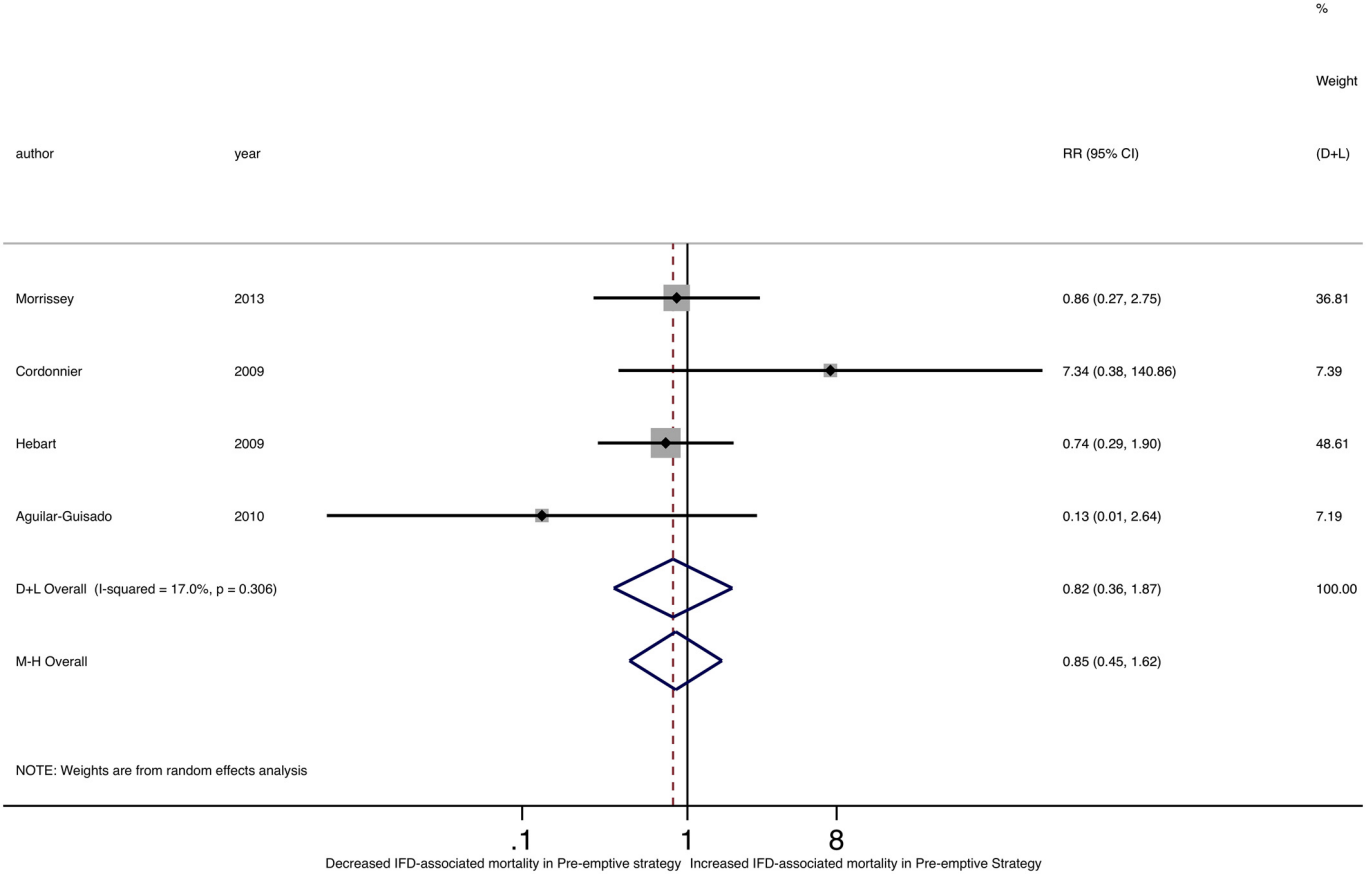
Forest plot of pooled relative risk of IFD-associated mortality comparing pre-emptive to empirical strategies.

**Fig 7:**
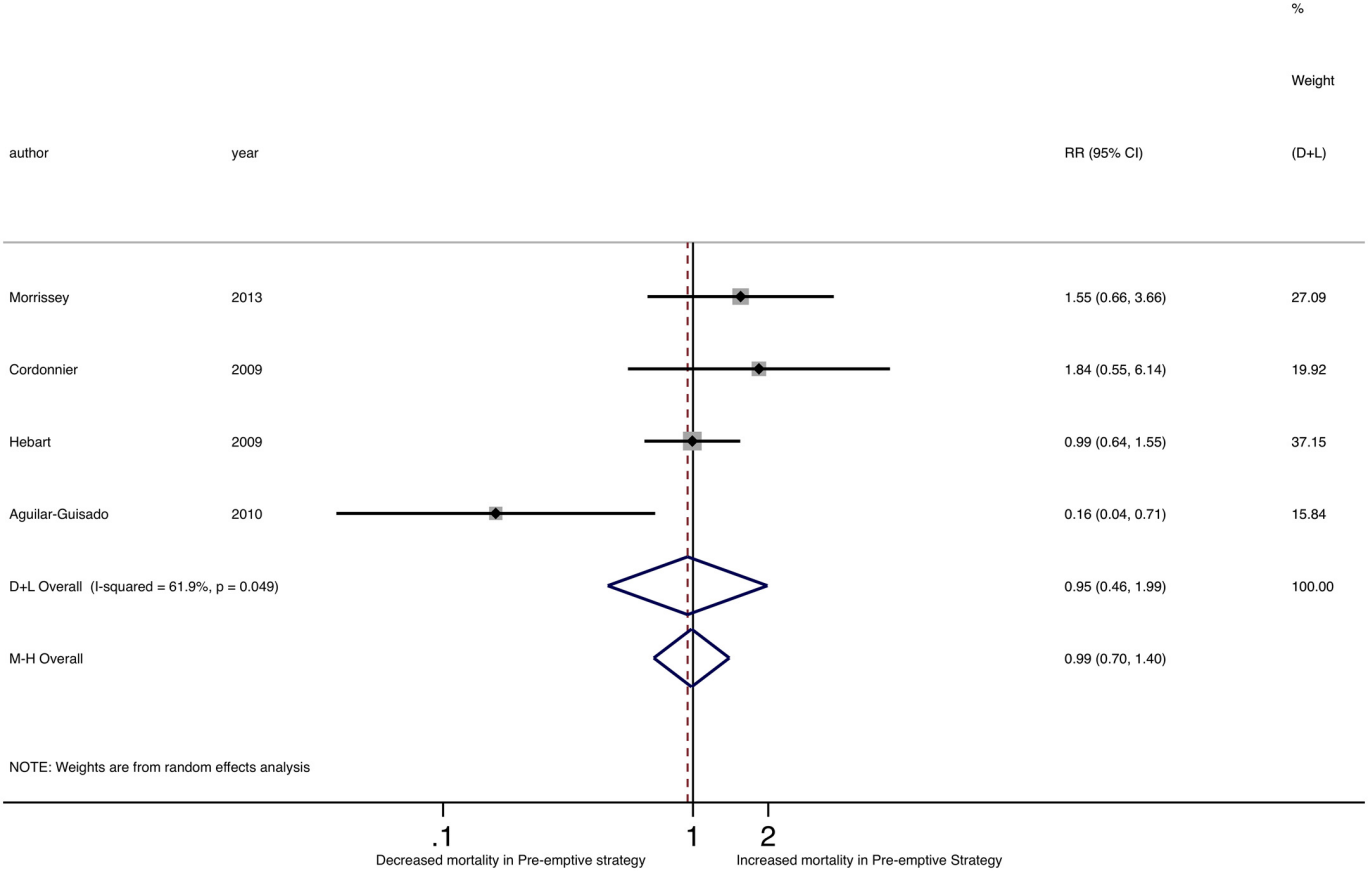
Forest plot of pooled relative risk of overall mortality comparing pre-emptive to empirical strategies.

Of the five studies reporting IFD-related mortality, none found a significant difference with empirical versus pre-emptive management (Fig 6) and there was no difference in IFD-related mortality pooled RR comparing the two groups (M-H RR 0.85, 95% CI 0.45–1.62; D-L RR 0.82, 95%CI 0.36–1.87). We found no significant heterogeneity among individual study RRs of IFD-related mortality (Cochran’s Q p = 0.306; I^2^ = 17.0%; τ^2^ = 0.132). While one study identified a significant difference in overall mortality between pre-emptive and empirical strategies, with decreased mortality with the pre-emptive strategy (RR 0.16, 95%CI 0.04–0.71)[36], there was no difference in the pooled RR of overall mortality between the two groups (M-H RR 0.99, 95%CI 0.70–1.40; D-L RR 0.95, 95%CI 0.46–1.99) (Fig 7). Of note, there was significant heterogeneity in overall mortality rates (Cochran’s Q p = 0.05; I^2^ = 61.9%; τ^2^ = 0.328).

**Fig 8:**
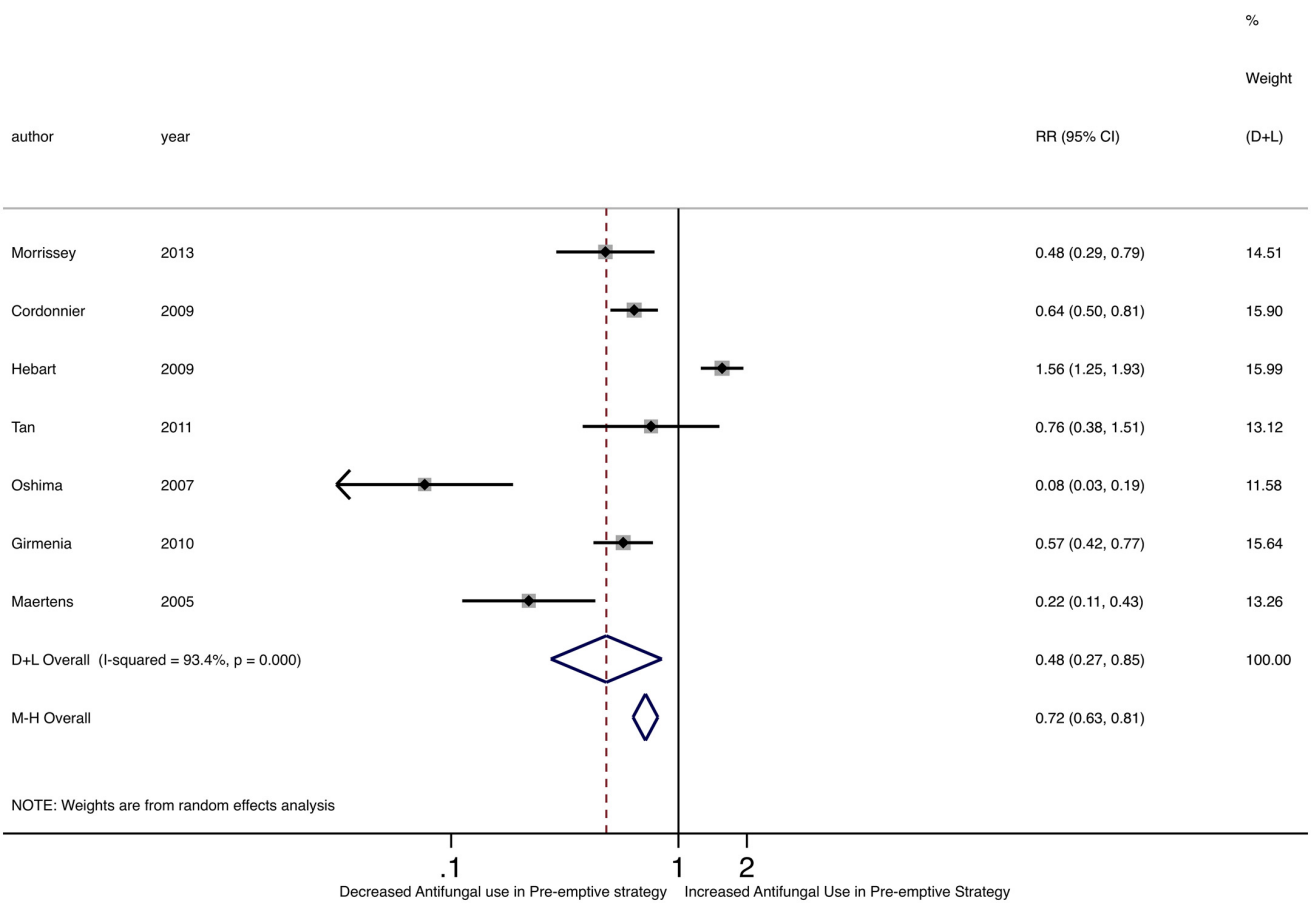
Forest plot of pooled relative risk of antifungal drug use comparing pre-emptive to empirical strategies.

Five of seven studies assessing antifungal use found a significant decrease in antifungal use in the pre-emptive therapy group (Table 3, Fig 8) [25,26,28–30]. For example, in the Cordonnier trial, 61.3% of patients on the empirical strategy arm received antifungals compared to 39.2% of patients on the pre-emptive strategy arm. Furthermore, among patients who did receive antifungals, the duration of antifungal use was shorter in the pre-emptive group [26], with an average antifungal duration of 7.0–11.2 days in the empirical group and 4.5–8.7 days in the pre-emptive group. Notably, there was no difference in antifungal drug toxicity, either renal or hepatic, or in discontinuation of antifungal therapy between the two groups in any of the studies. The pooled RR of antifungal use was significantly lower using pre-emptive compared to empirical strategies (M-H RR 0.72, 95%CI 0.63–0.81; D-L 0.48, 95%CI 0.27–0.85). Of note, we detected statistical heterogeneity among individual RRs of antifungal drug use (Cochran’s Q p<0.001; I^2^ = 93.4%; τ^2^ = 0.503).

Overall, the existing literature demonstrates that pre-emptive diagnostic-test driven approaches may increase IFD detection without an increase in IFD-related or overall mortality, compared to empirical therapy. The majority of studies showed a reduction in the proportion of patients receiving antifungals and duration of antifungal exposure using a pre-emptive strategy.

### Cost Comparison Model

Tables 4 and 5 outline clinical and cost parameters incorporated into the cost-comparison analysis, respectively, and Table 6 summarizes the results of this model. Assuming similar diagnostic strategies and otherwise similar baseline costs, the pre-emptive approach cost $325 less than the empirical approach overall ($2053.50 pre-emptive vs. $2378.00 empirical) per febrile neutropenic episode. During the empirical or pre-emptive period alone, empirical therapy cost $594 more than pre-emptive management ($1,209 pre-emptive vs. $1,803 empirical) per FN episode, due to a greater rate and duration of antifungal drug exposure using the empirical strategy.

**Table 4:** Clinical parameters incorporated into cost comparison model.

Clinical Data	Value	Reference
Duration of Neutropenia (days)	18 days	[26]
IFD detection		
Empirical Therapy	0.068	Table 2
Pre-emptive Therapy	0.100	Table 2
**Antifungal Use Data**		
Ratio of Pre-IFD diagnosis antifungal use	0.48	Table 3
Ratio of Pre-IFD diagnosis antifungal duration	0.64	Table 3
Empirical Therapy		
Proportion with empirical antifungal use	0.50	Table 3
Duration of empirical antifungal use (days)	7.0	[26]
Pre-emptive Therapy		
Proportion with pre-emptive antifungal use	0.24	Calculated ^a^
Duration of pre-emptive antifungal use (days)	4.5	Calculated ^b^
Duration of antifungal treatment	84 days	[24]

^a^[Proportion with antifungal treatment (empirical)] x [Ratio of antifungal treatment]

^b^[Antifungal duration (empirical)] x [Ratio of antifungal duration]

**Table 5:** Cost parameters incorporated into cost comparison model.

Diagnostic Testing Costs		Value ($)	Reference
Diagnostic Test			
Chest CT scan		414	[32]
Galactomannan test		133	BWH/DFCI^a^ Laboratory
Cost of Diagnostic Testing (per patient per week of persistent febrile neutropenia >4 days)			
Empirical Therapy	2 galactomannan/week and 1 CT scan	946	[26]
Pre-emptive Therapy	2 galactomannan/week and 1 CT scan	946	Expert opinion
**Antifungal Treatment Costs**			
Pre-IFD Diagnosis Antifungals (daily per 65kg patient)	(90% mica, 5% vori, 5% L-AmB)	244	Expert opinion
Incident IFD Treatment Antifungals (daily per 65kg patient)	(85% vori, 5% posa, 5% L-AmB, 5% mica)	101	Expert opinion
Liposomal amphotericin B (L-AmB)	3mg/kg at $196.25 per 50mg vial	785	[33]
Micafungin (mica)		224	[33]
Voriconazole (vori)	2x200mg tablets	49	[33]
Posaconazole (posa)	800mg/day	172	[33]

^a^Brigham and Women’s Hospital/Dana-Farber Cancer Institute

**Table 6:** Results of the cost-comparison model^a^.

Strategy	Pre-IFD Diagnosis Antifungals^b^	Incident IFD Treatment Antifungals^c^	Total Cost^d^ ($)
Empirical Therapy	858	574	2378
Pre-emptive Therapy	263	844	2053

^a^All costs are per patient.

^b^[Daily cost of pre-IFD diagnosis antifungals] x [Proportion with pre-IFD diagnosis antifungals] x [Duration of pre-IFD diagnosis antifungals]

^c^[Daily cost of incident IFD treatment antifungals] x [IFD detection] x [Duration of incident IFD treatment antifungals]

^d^[Cost of diagnostic testing] + [Cost of pre-IFD diagnosis antifungals] + [Cost of incident IFD treatment antifungals]

### Sensitivity Analysis

Our cost estimates did not change when we derived model parameters only from the most robust randomized controlled trials of empirical versus preemptive antifungal therapy [26,31]. See supplementary material for plug-in model allowing for base case parameter adjustments (S1 Model).

Probabilistic sensitivity analysis using 95% confidence intervals derived from our meta-analysis for relative risks of IFD detection and antifungal use resulted in 95% credible intervals for the cost of pre-emptive therapy being $291.88 less to $418.65 more costly than empirical therapy overall, and pre-emptive therapy being $269.00 less to $106.33 more costly than empirical therapy prior to IFD detection. With one-way sensitivity analysis, with the RR of IFD detection comparing pre-emptive and empirical groups increased to a threshold of RR of 2.03, pre-emptive therapy became the more costly strategy. As long as the proportion of patients in the pre-emptive group receiving antifungal treatment was less than or equal to the proportion of people being treated in the empirical therapy group, empirical therapy was always the more costly strategy.

Our results were sensitive to changes in the diagnostic testing strategy, antifungal drug costs, antifungal duration, and ratios of incident IFD diagnoses and antifungal use between the pre-emptive and empirical groups. For the diagnostic testing strategy, if the pre-emptive group received an additional chest CT scan per FN episode, the two strategies were nearly equivalent in cost with the empirical group being only $90 more costly overall per FN episode.

With regards to antifungal drugs, the pre-emptive strategy became equivalent to empirical therapy (more costly by $12 per patient) using the most conservative estimate of drug costs (voriconazole) at $49 per day. In contrast, using a more expensive drug regimen of liposomal amphotericin B ($785 per day) in the pre-IFD diagnosis period and posaconazole ($172 per day) in the post-IFD diagnosis period made empirical therapy the even more costly strategy by $1,453 per FN episode. At a threshold pre-IFD diagnosis antifungal drug cost of $111 per day and post-IFD diagnosis antifungal drug cost of $221 per day, the two strategies were equivalent in cost. At an antifungal treatment duration threshold of less than 185 days following incident IFD diagnosis, pre-emptive therapy became the more costly strategy.

## Discussion

This study provides the first systematic analysis of available evidence comparing empirical and pre-emptive antifungal therapy strategies among hematologic malignancy or HSCT patients with high-risk FN. Overall, pre-emptive therapy was associated with decreased antifungal treatment rates and duration and diagnostic-test driven increased IFD detection without an increase in IFD-related mortality. We incorporated these composite estimates in a cost comparison model and found a state of economic equipoise between empirical and pre-emptive antifungal therapy, slightly favoring pre-emptive therapy as the less costly strategy by $325 per FN episode, but influenced by relatively small changes in the cost of antifungal therapy and diagnostic testing. However, overall costs incorporating treatment for proven and probable IFD were consistently higher with pre-emptive therapy, largely due to the increased diagnosis of incident IFD and the cost of antifungal treatment for diagnosed IFD cases in this group.

The influence of diagnostic testing for IFD is evident in our results. Pre-emptive approaches in the studies we examined varied significantly in fungal biomarker and imaging use. In particular, studies we reviewed employed varying combinations of galactomannan, beta-D-glucan, and fungal PCR. Of these, serum galactomannan was the most common biomarker trigger for starting pre-emptive antifungal therapy. Fungal PCR was also used in conjunction with galactomannan in some of these studies, though this assay is subject to variations in internal and external validity [38]. Still, a recent study showed that galactomannan combined with Aspergillus PCR may lead to earlier diagnosis of invasive aspergillosis, lower IFD detection, and reduced antifungal use compared to galactomannan alone [39].

Also important to consider is the clinical and economic significance of IFD diagnoses in the setting of augmented diagnostic surveillance. Higher IFD detection without a corresponding increase in IFD-related mortality with pre-emptive therapy likely reflects the sensitivity of serial, systematically assessed fungal biomarkers and chest imaging for IFD, with initiation of antifungal therapy when there is relatively limited extent of fungal disease. However, higher rate of IFD identification with the pre-emptive strategy led to increased antifungal treatment and overall costs compared to the empirical group.

This work also highlights the heterogeneity of antifungal drug treatment for incident IFD. Pre-emptive management led to an overall reduction in antifungal use and duration. However, the cost of specific antifungal agents influenced which approach was more costly. This is particularly relevant given institutional and regional variability in IFD epidemiology, antifungal drug susceptibility, and clinical management of patients with prolonged FN. Specifically, combination antifungal therapy is increasingly used in refractory cases with a recent study suggesting potentially increased efficacy in the treatment of early invasive aspergillosis [40].

Limitations of our systematic review and cost comparison are largely intrinsic to the available literature comparing empirical and pre-emptive antifungal therapy in FN patients. Many of the studies we incorporated into our estimates of IFD detection, IFD-related mortality, antifungal use, and antifungal duration were small, observational studies [27,34]. Together, works we included encompassed heterogeneous patient groups with varying IFD prevalence, underlying immune deficits, antifungal prophylaxis rates, definitions of empirical and preemptive therapy, diagnostic testing strategies, and treatment regimens for incident IFD. To address this issue in part, we conducted tests of heterogeneity and, finding evidence of heterogeneity in some of these parameters between studies, used mixed effect models to estimate pooled relative risks. Sensitivity analysis using parameters derived from only the largest, most robust comparisons of empirical and pre-emptive antifungal therapy also did not change the outcome of our cost comparison model.

While simple, our cost comparison model incorporated key differences in antifungal use, diagnostic testing, and clinical outcomes driving cost differences between the two strategies. Compared to the two existing published cost-effectiveness studies comparing empirical and pre-emptive antifungal therapy in FN [22,23], our model parameters are derived from composite estimates using all available data comparing these two strategies. In contrast, Martín-Peña *et al* used parameters derived solely from two small observational studies and assumed significant differences in key patient characteristics and outcomes. While they found that pre-emptive therapy was 2.6% more effective and 33% less costly, their results may have limited generalizability and validity [22]. Similarly, Barnes *et al* constructed a model that made several assumptions (most notably, differences in IFD-associated mortality, overall mortality, and antifungal toxicity between empirical and pre-emptive strategies) that are not supported by current literature examining empirical versus pre-emptive antifungal therapy [23,26,29,30,31].

Overall, we show that a diagnostic test-guided pre-emptive approach to antifungal management in FN is a clinically and economically reasonable alternative to fever-based empirical therapy. Forthcoming results of the EORTC randomized trial of caspofungin for empirical versus diagnostic-guided pre-emptive antifungal therapy in patients with acute myeloid leukemia or myelodysplastic syndrome and the subsequent economic analysis should help further elucidate some of these issues substantially [41]. Our findings also emphasize the need for ongoing investigation of the utility and cost of existing fungal diagnostic tests and the development of more efficient, accurate, and cost-effective diagnostic strategies to shift economic momentum towards more rational pre-emptive therapeutic approaches.

## Supporting Information

S1 Dataset Original study data incorporated into meta-analysis. (XLSX)

S1 File PRISMA Checklist. (DOC)

S1 Model Plug-in model allowing for base case parameter adjustments. (XLS)
